# Dexmedetomidine Alleviates Microglia-Induced Spinal Inflammation and Hyperalgesia in Neonatal Rats by Systemic Lipopolysaccharide Exposure

**DOI:** 10.3389/fncel.2021.725267

**Published:** 2021-12-08

**Authors:** Wen Wen, Xingrui Gong, Hoiyin Cheung, Yanyan Yang, Meihua Cai, Jijian Zheng, Xiaoping Tong, Mazhong Zhang

**Affiliations:** ^1^Department of Anesthesiology, Shanghai Children’s Medical Center, Shanghai Jiao Tong University School of Medicine, Shanghai, China; ^2^Center for Brain Science of Shanghai Children’s Medical Center, Shanghai Jiao Tong University School of Medicine, Shanghai, China; ^3^Department of Anesthesiology, Xiangyang Central Hospital, Hubei University of Arts and Science, Xiangyang, China; ^4^Department of Anesthesiology, Ruijin Hospital, Shanghai Jiao Tong University School of Medicine, Shanghai, China; ^5^Department of Anatomy and Physiology, Shanghai Jiao Tong University School of Medicine, Shanghai, China

**Keywords:** lipopolysaccharide-induced inflammation, spinal cord, hyperalgesia, microglia, dexmedetomidine

## Abstract

Noxious stimulus and painful experience in early life can induce cognitive deficits and abnormal pain sensitivity. As a major component of the outer membrane of gram-negative bacteria, lipopolysaccharide (LPS) injection mimics clinical symptoms of bacterial infections. Spinal microglial activation and the production of pro-inflammatory cytokines have been implicated in the pathogenesis of LPS-induced hyperalgesia in neonatal rats. Dexmedetomidine (DEX) possesses potent anti-neuroinflammatory and neuroprotective properties through the inhibition of microglial activation and microglial polarization toward pro-inflammatory (M1) phenotype and has been widely used in pediatric clinical practice. However, little is known about the effects of DEX on LPS-induced spinal inflammation and hyperalgesia in neonates. Here, we investigated whether systemic LPS exposure has persistent effects on spinal inflammation and hyperalgesia in neonatal rats and explored the protective role of DEX in adverse effects caused by LPS injection. Systemic LPS injections induced acute mechanical hyperalgesia, increased levels of pro-inflammatory cytokines in serum, and short-term increased expressions of pro-inflammatory cytokines and M1 microglial markers in the spinal cord of neonatal rats. Pretreatment with DEX significantly decreased inflammation and alleviated mechanical hyperalgesia induced by LPS. The inhibition of M1 microglial polarization and microglial pro-inflammatory cytokines expression in the spinal cord may implicate its neuroprotective effect, which highlights a new therapeutic target in the treatment of infection-induced hyperalgesia in neonates and preterm infants.

## Introduction

Neonates and preterm infants in neonatal intensive care units often undergo various invasive and painful procedures, such as intravenous cannulation, adhesive removal, fingerstick, and venous or arterial puncture (Chen et al., 2012). Noxious stimulus and painful experience in early life can induce cognitive deficits and abnormal pain sensitivity occurrence later in life (Boisse et al., 2005; Hermann et al., 2006; Wang et al., 2011; Ranger et al., 2015; Nuseir et al., 2017).

Neonates and infants also have a high incidence of infection (Rudd et al., 2020). An infection can cause hyperalgesia (Ji et al., 2016), which makes neonates and infants more sensitive and respond more exaggeratedly to painful procedures. As a major component of the outer membrane of gram-negative bacteria, lipopolysaccharide (LPS) injection mimics clinical symptoms of bacterial infections, such as systemic inflammatory responses and hyperalgesia (Wegner et al., 2014, 2015). Meanwhile, LPS injection has been reported to evoke a persistent pro-inflammatory reactions in the brain, which may correlate with abnormal function in the central nervous system (CNS) (Gisslen et al., 2019).

Microglia can be both detrimental and beneficial in CNS disorders (Gomes-Leal, 2012). Upon appropriate stimulation, microglia can polarize toward pro-inflammatory (M1) phenotype and anti-inflammatory (M2) phenotype. The M1 phenotype can release pro-inflammatory cytokines, while the M2 phenotype can secrete anti-inflammatory cytokines to attenuate pro-inflammatory responses (Marques et al., 2004; Orihuela et al., 2016). Spinal microglia play a key role in central sensitization and hyperalgesia in which the mechanism involves microglial polarization toward an M1 phenotype and the production of pro-inflammatory cytokines to facilitate pain transmission in the spinal cord (Kawasaki et al., 2008; Ji et al., 2016; Gong et al., 2017). Therefore, spinal microglial activation and inflammation have been implicated in the pathogenesis of LPS-induced hyperalgesia (Hsieh et al., 2018). Meanwhile, decreased hyperalgesia has been accompanied with the upregulation of microglial M2 phenotype and increased levels of anti-inflammatory cytokines in the spinal cord (Gong et al., 2017; Huo et al., 2018).

Neutrophils are one of the types of leukocytes in the blood. After brain damage, neutrophils can infiltrate the CNS (Carlos et al., 1997). It has been reported that neutrophils are one of the sources of pro-inflammatory cytokines and that they may contribute to CNS inflammation (Ji et al., 2007; Steinbach et al., 2013). They have also been found in the brain after systemic LPS injection (Zhou et al., 2009; Wu et al., 2020).

Dexmedetomidine, a mainly selective adrenergic α2 receptor agonist, has been widely used in pediatric clinical practice for sedation (Mahmoud et al., 2020). It has been reported that dexmedetomidine (DEX) displays an anti-neuroinflammatory and neuroprotective property, possibly through the inhibition of microglial activation and microglial polarization toward the M1 phenotype (Yeh et al., 2018; Gao et al., 2019). Moreover, DEX can induce M2 microglial polarization and increase levels of microglia-associated anti-inflammatory cytokines (Sun et al., 2018; Gao et al., 2019; Qiu et al., 2020), which may also implicate its anti-inflammatory effect. Nevertheless, it remains unclear whether DEX can attenuate inflammation and hyperalgesia triggered by systemic LPS injection during the neonatal period.

In this study, we examined whether systemic LPS exposure has a persistent effect on spinal inflammation and hyperalgesia in neonatal rats and further estimated the protective role of DEX in adverse effects caused by LPS injection. We found that systemic LPS injection-induced acute mechanical hyperalgesia in neonatal rats. Systemic inflammation, short-term pro-inflammatory cytokine production, and microglial activation in the spinal cord were also observed in neonatal rats that underwent LPS injection. Pretreatment with DEX significantly decreased inflammation and alleviated mechanical hyperalgesia induced by LPS. The inhibition of M1 microglial polarization and microglial pro-inflammatory cytokine expression in the spinal cord may implicate the neuroprotective effect of DEX.

## Materials and Methods

### Animals

The study was approved by the Laboratory Animal Welfare and Ethics Committee of Shanghai Children’s Medical Center (approval number SCMC-LAWEC-2019-0016), an affiliate of Shanghai Jiao Tong University School of Medicine, and was conducted under the guidelines of the National Institute of Health. Sprague–Dawley rats were purchased from Charles River (Beijing, China). Pregnant female Sprague–Dawley rats were obtained and kept in a room with constant temperature and humidity. All the animals were maintained on a 12-h light/dark cycle (lights on between 7:00 and 19:00 h) and had free access to food and water. After birth, rat pups were sexed, and only males were included in this study. A total of about 300 neonatal rats were used in this study.

### Drugs

Lipopolysaccharide (from *Escherichia coli*, serotype 055: B5) was purchased from Sigma-Aldrich (United States). DEX was purchased from Yangtze River Pharmacy Group (Taizhou, China).

### Animal Treatment

To explore the effects of LPS on nociceptive behavior, inflammation, and microglial activation, postnatal day 5–6 (P5-6) rat pups were intraperitoneally injected with saline (total volume of 0.1 ml) or LPS (2 mg/kg). The dose of LPS was determined according to previous studies (Hsieh et al., 2018, 2020). Baseline mechanical threshold and thermal latency were measured 1 h before saline or LPS injection. Eight, 24, and 48 h after LPS injection, the rat pups either underwent a nociceptive behavior test or were sacrificed for mRNA analyses, enzyme-linked immunosorbent assay (ELISA), and immunofluorescence.

To explore the protective role of DEX in adverse effects caused by LPS injection, the P5-6 rat pups underwent an intraperitoneal injection of saline (total volume of 0.1 ml) or DEX (25 μg/kg). The dose of DEX was based on previous reports (Paeschke et al., 2017; Yeh et al., 2018). The baseline mechanical threshold was measured 1 h before saline or DEX injection. Thirty minutes after injection, the rat pups were intraperitoneally injected with saline (total volume of 0.1 ml) or LPS (2 mg/kg). They underwent a nociceptive behavior test 8, 24, and 48 h after the LPS injection, or were sacrificed for mRNA analyses and ELISA 8 h after LPS injections.

### Behavior Test

The rats were placed in an acrylic box on a mesh floor 50 cm above the table to test for mechanical hyperalgesia. After 5 min of acclimation, von Frey filaments (North Coast Medical, United States) were used to stimulate the plantar surface of one hindpaw. Mechanical threshold was defined as the von Frey filament that evoked paw withdrawal reflex in three out of five applications (Hsieh et al., 2018).

For thermal latency, the rats were habituated to the glass surface for 5 min and the time for withdrawal from a heat stimulus directed at the plantar surface of the hind paw was recorded. The heat stimulus was elicited using a plantar analgesia tester (Institute of Biomedical Engineering, CAMS, China). The mean of three measures was designated as thermal latency (Gong et al., 2016).

### RNA Extraction and RT-qPCR

All the animals were sacrificed in deep anesthesia with isoflurane. For total RNA extraction of tissue, the lumbar enlargement of the spinal cord was removed and homogenized with Trizol (Life Technologies, United States). The total RNA was subjected to reverse transcription using PrimeScript^TM^ RT reagent Kit with gDNA Eraser (Takara, Japan), followed by qPCR using Green^TM^ Premix Ex Taq^TM^ II (Takara, Japan). For RNA extraction of sorted microglia, microglia were homogenized with Trizol (Life Technologies, United States). Then, the RNA was subjected to dissolution, denaturation, reverse transcription, and amplification as described (Chen et al., 2017). qPCR was performed using Green^TM^ Premix Ex Taq^TM^ II (Takara, Japan). The primers are shown in Table 1. Glyceraldehyde 3-phosphate dehydrogenase (GAPDH) functioned as the endogenous control gene. Messenger RNA (mRNA) expressions were analyzed according to the 2^–ΔΔCT^ method.

**TABLE 1:** Primer sequences used for quantitative reverse transcription PCR (RT-qPCR).

	**5′–3′**
iNOS	CAGGCTTGGGTCTTGTTAGC
	TGTTGTTGGGCTGGGAATAG
CD86	AAGACATGTGTAACCTGCACC
	AGAACCGACTTTTTCCGGTC
IL-1β	CTGTGACTCGTGGGATGATG
	ACAGGGATTTTGTCGTTGCT
TNF-α	ATCGTCTACTCCTCAGAGCC
	ATCCAGTGAGTTCCGAAAGC
IL-4	CGGTATCCACGGATGTAACG ACTTGTTCTTCAAGCACGGA
IL-10	ACGCTGTCATCGATTTCTCC TGTCCTGCAGTCCAGTAGAT
GAPDH	CTCTGCTCCTCCCTGTTCTA
	GGTCAATGAAGGGGTCGTTG

### Enzyme-Linked Immunosorbent Assay

The lumbar enlargement of the spinal cord and blood were collected. The blood was allowed to clot for 30 min at room temperature. Then, it was centrifuged at 1,000 × *g* for 20 min at 4°C. Supernatants were subsequently collected as serum. Tissues were homogenized with PBS and centrifuged at 12,000 × *g* for 30 min at 4°C. The supernatant was collected, and total protein concentration was measured using BCA Protein Assay Kit (Beyotime Biotechnology, China). Cytokine levels were determined using ELISA kits (R&D Systems, United States). Cytokine contents were expressed as picograms of cytokines per milliliter of serum or picograms of cytokines per milligram of protein.

### Immunofluorescence

The rats were perfused with saline followed by 4% paraformaldehyde. The lumbar enlargement of the spinal cord was removed and fixed with 4% paraformaldehyde at 4°C, followed by 30% sucrose for cryoprotection overnight. The lumbar spinal cord sections between L4-L5 were then cut into 25-μm thickness with a cryostat (Leica, Germany) and incubated with the following primary antibodies overnight: polyclonal rabbit anti-Iba1 (1:500; Wako Chemistry, Japan) and polyclonal rabbit anti-MPO (1:250; Abcam, Britain). The sections were further incubated with secondary antibody goat anti-rabbit for Alexa Fluor 594 (1:1,000; Abcam, Britain) for 2 h at room temperature. 4′,6-Diamidino-2-phenylindole (DAPI) (Beyotime Biotechnology, China) was used to stain nuclei. The sections were covered and examined using a confocal microscope (Leica, Germany).

### Quantitative Analysis of Microglia and Neutrophils

For the measurement of cell numbers, a microscopic image of the spinal dorsal horn region was captured in each of the two or three sections, and the number of Iba1 or MPO positively immunolabeled cells in each section was counted and averaged (cells/mm^2^). The mean value of cell counting from the spinal cord sections was used to represent one sample of the spinal cord. For the measurement of the soma size of microglia, eight Iba1 positively immunolabeled cells in each section were selected, and their soma sizes were measured and averaged. The mean value of soma sizes from spinal cord sections represents one sample of the spinal cord. The analysis of cell numbers and soma sizes was performed using the Image J software (NIH, United States), and the tissue sections were from four animals in each group.

### Sorting of Microglia

The microglia were sorted by using fluorescence-activated cell sorting. The rats were perfused with PBS, and the lumbar enlargement of the spinal cord was harvested. The tissues were dissected into small pieces and digested with trypsin (Life Technologies, United States) for 30 min at 37°C. The cell suspension was filtered and centrifuged at 1,500 rpm for 5 min at 4°C. The cell pellets were washed and re-suspended in phosphate-buffered saline (PBS). Then, the cells were stained with fluorescein isothiocyanate-conjugated mouse anti-rat CD45 antibody (1:800; BD Pharmingen, United States) and allophycocyanin-conjugated mouse anti-rat CD11b antibody (1:320, BD Pharmingen, United States) for 30 min at 4°C. An unstained antibody was washed in PBS. DAPI (1:10,000; Beyotime Biotechnology, China) was used to discriminate live/dead cells. The microglia were identified as CD45^*low*^CD11b^+^cells and gathered with a Moflo XDP flow cytometry sorter (Beckman Coulter, United States).

### Statistical Analysis

The statistical analyses were performed using Prism 8.0 (GraphPad Software, United States), and data were presented as the mean ± SEM. The results from the behavioral test were analyzed through two-way repeated-measures ANOVA followed by Bonferroni’s multiple comparisons test. The results from quantitative reverse transcription PCR (RT-qPCR) were analyzed by unpaired *t*-test, Mann–Whitney test, Brown–Forsythe and Welch ANOVA test, Kruskal–Wallis test or one-way ANOVA. The results from ELISA were analyzed by unpaired *t*-test, one-way ANOVA or Brown-Forsythe and Welch ANOVA tests. The results from immunostaining were analyzed by unpaired *t*-test. Results with *P* < 0.05, 0.01, 0.001, or 0.0001 were considered statistically significant.

## Results

### Systemic Injection of Lipopolysaccharide Induced Acute Mechanical Hyperalgesia but Not Thermal Hyperalgesia in Neonatal Rats

**FIGURE 1:**
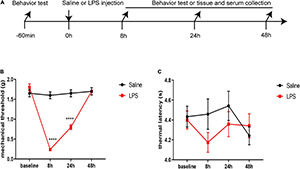
Lipopolysaccharide (LPS) induced acute mechanical hyperalgesia but not thermal hyperalgesia. **(A)** Experimental design. P5-6 rat pups were intraperitoneally injected with saline or LPS. Baseline mechanical threshold and thermal latency were measured 1 h before saline or LPS injection. Eight, 24, and 48 h after injection, rat pups underwent a nociceptive behavior test or were sacrificed for messenger ribonucleic acid (mRNA) analyses, ELISA, and immunofluorescence. **(B)** Rats in the LPS group showed decreased mechanical threshold 8 and 24 h after LPS injection. **(C)** No significant difference was found in thermal latency between the rats in the LPS group and the rats in the Saline group at each time point. The results are expressed as the mean ± standard error of the mean (SEM), *n* = 12 animals for each group and were analyzed by two-way repeated-measures analysis of variance (ANOVA) followed by Bonferroni’s multiple comparisons test. ****P < 0.0001 LPS group vs. Saline group.

To study the effect of systemic injection of LPS on nociceptive behavior, the mechanical threshold and thermal latency were observed 8, 24, and 48 h after LPS injection (Figure 1A). There was no significant difference in the baseline of mechanical threshold and thermal latency between the two groups (*P* > 0.05). However, we found that rats in the LPS group showed a significant decrease in mechanical threshold 8 and 24 h after LPS injection compared with those in the saline group (8 h: 0.24 ± 0.03 vs. 1.6 ± 0.09, *P* < 0.0001; 24 h: 0.8 ± 0.06 vs. 1.65 ± 0.09, *P* < 0.0001) (Figure 1B). Forty-eight hours after LPS injection, there was no significant difference in mechanical threshold between rats in the LPS group and rats in the saline group (*P* > 0.05). There was no significant difference in the thermal latency between the LPS and saline groups at each time point (*P* > 0.05) (Figure 1C). In summary, LPS induced acute mechanical hyperalgesia but not thermal hyperalgesia in neonatal rats.

### Systemic Injection of Lipopolysaccharide Induced Increased Levels of Pro-Inflammatory Cytokines in Both Serum and Spinal Cord of Neonatal Rats

**FIGURE 2:**
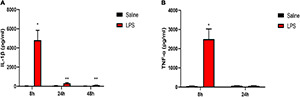
LPS induced increased levels of pro-inflammatory cytokines in serum. **(A)** Serum level of IL-1β was elevated 8, 24, and 48 h after LPS injection in neonatal rats. **(B)** Serum level of TNF-α was elevated 8 h after LPS injection in neonatal rats. Results are expressed as the mean ± SEM, *n* = 4–5 animals for each group, and were analyzed by unpaired *t*-test. *P < 0.05, **P < 0.01 LPS group vs. Saline group.

We first examined the protein levels of pro-inflammatory cytokines interleukin-1beta (IL-1β) and tumor necrosis factor-alpha (TNF-α) in the serum 8, 24, and 48 h after LPS injection. There was a dramatic increased level of IL-1β at 8, 24, and 48 h after LPS injection compared with that in the saline group (8 h: 4,807.71 ± 1,027.01 vs. 30.23 ± 13.1, *P* < 0.05; 24 h: 325.24 ± 66.67 vs. 48.46 ± 23.49, *P* < 0.01; 48 h: 101.7 ± 19.29 vs. 24.45 ± 6.87, *P* < 0.01) (Figure 2A). Meanwhile, a significantly increased level of TNF-α 8 h after LPS injection was also observed compared with that in the saline group (2,497.7 ± 530.95 vs. 44.14 ± 9.43, *P* < 0.05) (Figure 2B).

**FIGURE 3:**
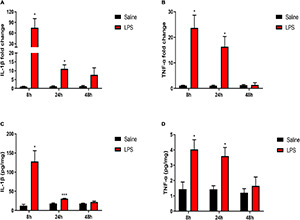
LPS increased levels of pro-inflammatory cytokines in the spinal cord. **(A,B)** mRNA levels of IL-1β and TNF-α were elevated 8 and 24 h after LPS injection in neonatal rats. **(C,D)** Protein expressions of IL-1β and TNF-α were elevated 8 and 24 h after LPS injection in neonatal rats. Results are expressed as the mean ± SEM, *n* = 4–5 animals for each group, and were analyzed by unpaired *t*-test or Mann–Whitney test. *P < 0.05, ***P < 0.001 LPS group vs. Saline group.

The mRNA and protein levels of pro-inflammatory cytokines IL-1β and TNF-α in the spinal cord were also examined 8, 24, and 48 h after LPS injection. The rats in the LPS group showed significantly increased mRNA levels of IL-1β and TNF-α 8 and 24 h after LPS injection compared with the rats in the saline group (8 h IL-1β: 74.85 ± 26 vs. 1.08 ± 0.2, *P* < 0.05; 8 h TNF-α:23.72 ± 4.94 vs. 1.1 ± 0.17, *P* < 0.05; 24 h IL-1β: 11.05 ± 2.31 vs. 1.2 ± 0.3, *P* < 0.05; 24 h TNF-α:16.34 ± 3.99 vs. 1.1 ± 0.19, *P* < 0.05) (Figures 3A,B). In addition, a significant increase in the protein expression of IL-1β 8 and 24 h after LPS injection was also observed in the LPS group compared with the Saline group (8 h: 127.97 ± 28.11 vs. 12.51 ± 3.56, *P* < 0.05; 24 h: 30.9 ± 1.02 vs. 18.18 ± 1.64, *P* < 0.001) (Figure 3C). Meanwhile, the rats showed a significant increase in the protein expression of TNF-α 8 and 24 h after LPS injection compared with rats in the saline group (8 h: 4.03 ± 0.63 vs. 1.43 ± 0.47, *P* < 0.05; 24 h: 3.6 ± 0.56 vs. 1.43 ± 0.24, *P* < 0.05) (Figure 3D). Forty-eight hours after LPS injection, there were no significant differences in the mRNA and protein expressions of pro-inflammatory cytokines IL-1β and TNF-α between the LPS group and the Saline group (*P* > 0.05). Taken together, short-term inflammation in the spinal cord was induced by systemic injection of LPS in neonatal rats.

### Systemic Injection of Lipopolysaccharide Induced Microglial Activation in the Spinal Cord of Neonatal Rats

**FIGURE 4:**
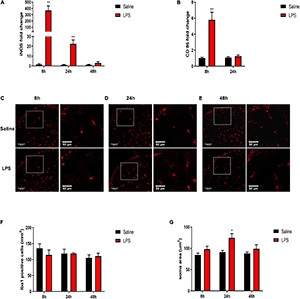
Effects of systemic injection of LPS on M1 microglial markers, microglial number, and morphology in the spinal cord of neonatal rats. **(A)** mRNA level of iNOS was elevated 8 and 24 h after LPS injection in neonatal rats. **(B)** mRNA level of CD86 was elevated 8 h after LPS injection in neonatal rats. **(C–E)** Representative photomicrographs of Iba1 immunostaining in the rat spinal cord dorsal horn 8, 24, and 48 h after saline or LPS injection. Scale bars, 100 and 50 μm for the inset images. **(F)** Systemic LPS exposure did not change Iba1-positive cell numbers 8, 24, and 48 h after LPS injection. **(G)** Systemic LPS exposure induced enlarged soma size of Iba1-positive microglia 24 h after injection. Results are expressed as the mean ± SEM, *n* = 4–5 animals for each group, and were analyzed by unpaired *t*-test or Mann–Whitney test. *P < 0.05, **P < 0.01. LPS group vs. Saline group.

Upon stimulation, microglia can polarize toward the pro-inflammatory (M1) phenotype and release pro-inflammatory cytokines (Orihuela et al., 2016; Li J. et al., 2020). Therefore, we directly examined the mRNA expressions of M1 microglial marker-inducible NO synthase (iNOS) and CD86. It showed significantly increased expressions of iNOS and CD86 8 h after LPS injection compared with that in the saline group (iNOS: 367.74 ± 72.4 vs. 1.59 ± 0.82, *P* < 0.01; CD86: 5.8 ± 0.94 vs. 1.02 ± 0.1, *P* < 0.01, Figures 4A,B). Meanwhile, the expression of iNOS was significantly elevated 24 h after LPS injection compared with that in the saline group (22.59 ± 3.86 vs. 1.15 ± 0.29, *P* < 0.01, Figure 4A), although the iNOS expression level returned back to the control level 48 h after LPS injection (*P* > 0.05). Besides the changes in inflammatory factors, microglia response to a noxious stimulus also includes proliferation and morphological changes (a transition from ramified to amoeboid shapes with enlarged cell bodies and shortened processes) (Chen et al., 2018). Thus, the effects of LPS exposure on microglial cell number and morphology in the dorsal horn of the spinal cord, as indicated by Iba1 immunofluorescence, were determined. In short, there was no overall change in the number of Iba1-positive cells between the saline group and the LPS group 8, 24, and 48 h post injection (*P* > 0.05) (Figures 4C–F). However, Iba1-positive microglia showed an enlarged soma size 24 h after LPS injection (124.5 ± 9.89 vs. 91 ± 4.02, *P* < 0.05) (Figures 4D,G) but not 8 or 48 h post injection of LPS (*P* > 0.05) (Figures 4C,E,G). Taken together, the results of increased mRNA expressions of M1 microglial markers and enlarged soma size in microglial morphology indicated the activation of microglia upon stimulation induced by LPS.

### Dexmedetomidine Pretreatment Attenuated Systemic Lipopolysaccharide-Induced Elevations of Pro-Inflammatory Cytokines and M1 Microglial Markers in the Spinal Cord of Neonatal Rats

**FIGURE 5:**
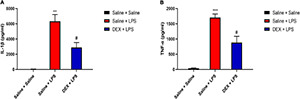
Dexmedetomidine (DEX) pretreatment attenuated systemic LPS-induced peripheral inflammation. **(A)** LPS induced an increased level of IL-1β in serum. DEX pretreatment decreased LPS-induced upregulation of IL-1β. **(B)** LPS induced an increased level of TNF-α in serum. DEX pretreatment decreased LPS-induced upregulation of TNF-α. Results are expressed as the mean ± SEM, *n* = 4–5 animals for each group, and were analyzed by Brown–Forsythe and Welch ANOVA tests followed by Tamhane’s T2 multiple comparisons test. **P < 0.01, ***P < 0.001 Saline + LPS group vs. Saline + Saline group. ^#^*P* < 0.05 DEX + LPS group vs. Saline + LPS group.

As DEX displays an anti-inflammatory and neuroprotective property (Li R. et al., 2020), we, thus, investigated whether DEX plays a role in the protection of neonatal rats from anti-inflammation and its possible mechanism. We found that consistent with the previous result, the rats in the Saline + LPS group showed significantly increased levels of IL-1β and TNF-α in the serum compared with the rats in the saline + saline group (IL-1β: 6,316.41 ± 896.03 vs. 38.81 ± 11.87, *P* < 0.01; TNF-α: 1,702.62 ± 118.48 vs. 36.53 ± 11.94, *P* < 0.001). However, DEX pretreatment largely decreased the serum levels of IL-1β and TNF-α (IL-1β: 2,883.23 ± 650.13 vs. 6,316.41 ± 896.03, *P* < 0.05; TNF-α: 884.01 ± 203.7 vs. 1,702.62 ± 118.48, *P* < 0.05, Figures 5A,B).

**FIGURE 6:**
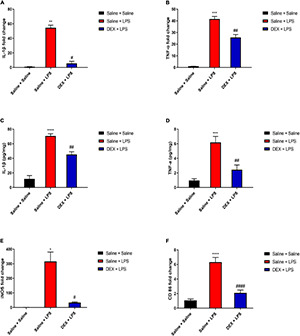
DEX pretreatment attenuated LPS-induced inflammation and expressions of M1 microglial markers in the spinal cord. **(A,B)** LPS induced increased mRNA levels of IL-1β and TNF-α. DEX pretreatment decreased LPS-induced mRNA levels of IL-1β and TNF-α. **(C,D)** LPS induced increased protein expressions of IL-1β and TNF-α. DEX pretreatment decreased LPS-induced protein expressions of IL-1β and TNF-α. **(E,F)** LPS induced increased mRNA levels of iNOS and CD86. DEX pretreatment decreased LPS-induced mRNA levels of iNOS and CD86. Results are expressed as the mean ± SEM, *n* = 4–6 animals for each group, and were analyzed by Kruskal–Wallis test followed by Dunn’s multiple comparisons test, Brown–Forsythe and Welch ANOVA tests followed by Tamhane’s T2 multiple comparisons test or one-way ANOVA followed by Tukey’s multiple comparisons test. *P < 0.05, **P < 0.01, ***P < 0.001, ****P < 0.0001 Saline + LPS group vs. Saline + Saline group. ^#^*P* < 0.05, ^##^*P* < 0.01, ^####^*P* < 0.0001 DEX + LPS group vs. Saline + LPS group.

We further tested the DEX protective effect in the spinal cord. As shown in Figures 6A,B, rats in the saline + LPS group showed significantly increased mRNA expressions of IL-1β and TNF-α compared with rats in the saline + saline group (IL-1β: 54.60 ± 3.4 vs. 1.05 ± 0.12, *P* < 0.01; TNF-α:41.58 ± 2.18 vs. 1.01 ± 0.06, *P* < 0.001). In contrast, DEX pretreatment largely decreased the mRNA expressions of IL-1β and TNF-α (IL-1β: 5.64 ± 2.85 vs. 54.60 ± 3.4, *P* < 0.05; TNF-α: 25.72 ± 2.6 vs. 41.58 ± 2.18, *P* < 0.01). In addition, rats in Saline + LPS group showed significantly increased protein expressions of IL-1β and TNF-α compared with rats in the Saline + Saline group (IL-1β: 70.66 ± 3.07 vs. 11.84 ± 4.56, *P* < 0.0001; TNF-α: 6.19 ± 0.82 vs. 0.96 ± 0.25, *P* < 0.001). DEX pretreatment significantly reduced the protein expressions of IL-1β and TNF-α (IL-1β: 45.34 ± 3.73 vs. 70.66 ± 3.07, *P* < 0.01; TNF-α: 2.43 ± 0.66 vs. 6.19 ± 0.82, *P* < 0.01) (Figures 6C,D). To test whether the reduction of inflammation cytokines is through the inhibition of M1 microglial polarization by DEX treatment, we found that, indeed, DEX pretreatment significantly reduced the mRNA expressions of iNOS and CD86, which are reported as M1 microglial markers (iNOS: 33.9 ± 4.26 vs. 315.71 ± 64.58, *P* < 0.05; CD86: 2.09 ± 0.4 vs. 6.32 ± 0.64, *P* < 0.0001) (Figures 6E,F). Taken together, our results indicate that DEX pretreatment alleviated LPS-induced spinal inflammation, which might be through the inhibition of M1 microglial polarization.

### Dexmedetomidine Pretreatment Attenuated Systemic Lipopolysaccharide-Induced Elevations of Pro-Inflammatory Cytokines in Sorted Microglia in the Spinal Cord of Neonatal Rats

**FIGURE 7:**
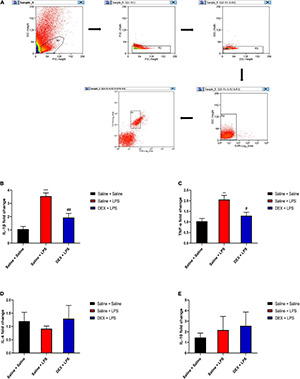
DEX pretreatment attenuated the LPS-induced expressions of pro-inflammatory cytokines in microglia. **(A)** The representative sorting strategy of microglia from lumbar enlargement of the spinal cord in neonatal rats. Microglia were considered as CD45^*low*^CD11b^+^ cells in the R5 area and were gathered for mRNA analysis. **(B,C)** LPS induced increased mRNA levels of IL-1β and TNF-α. DEX pretreatment decreased LPS-induced mRNA levels of IL-1β and TNF-α. **(D,E)** Neither LPS nor DEX induced significant changes in the mRNA expressions of IL-10 and IL-4. Results are expressed as the mean ± SEM, *n* = 4 animals for each group, and were analyzed by one-way ANOVA followed by Tukey’s multiple comparisons test or Kruskal–Wallis test followed by Dunn’s multiple comparisons test.**P < 0.01, ***P < 0.001, Saline + LPS group vs. Saline + Saline group. ^#^*P* < 0.05, ^##^*P* < 0.01, DEX + LPS group vs. Saline + LPS group.

To further demonstrate the effects of DEX on the expressions of inflammatory cytokines in microglia, we assessed the mRNA expressions in sorted microglia from the lumbar enlargement of the spinal cord which the sorting strategy was shown in Figure 7A. The microglia were identified as CD45^*low*^CD11b^+^cells as previously described (Zhang et al., 2002). As shown in Figures 7B,C, rats in the Saline + LPS group showed significantly increased mRNA expressions of IL-1β and TNF-α compared with rats in the Saline + Saline group (IL-1β: 3.55 ± 0.25 vs. 1.06 ± 0.21, *P* < 0.001; TNF-α: 2.06 ± 0.19 vs. 1.03 ± 0.13, *P* < 0.01). In contrast, DEX pretreatment significantly decreased the mRNA expressions of IL-1β and TNF-α (IL-1β: 1.93 ± 0.32 vs. 3.55 ± 0.25, *P* < 0.01; TNF-α: 1.29 ± 0.17 vs. 2.06 ± 0.19, *P* < 0.05). As it has been reported that the anti-inflammatory effects of DEX may involve the induction of M2 microglial polarization and expressions of anti-inflammatory cytokines in microglia (Gao et al., 2019; Qiu et al., 2020), we further evaluated the mRNA expressions of anti-inflammatory cytokines interleukin-10 (IL-10) and interleukin-4 (IL-4) in sorted microglia as well. As shown in Figures 7D,E, however, we did not detect a significant difference in the expressions of IL-10 and IL-4 between the rats in the Saline + Saline group and the rats in the Saline + LPS group (*P* > 0.05), suggesting a major role of M1 microglia in LPS-induced spinal inflammation. Meanwhile, there was no significant difference in the expressions of IL-10 and IL-4 between the rats in the Saline + LPS group and the rats in the DEX + LPS group (*P* > 0.05) (Figures 7D,E).

### Dexmedetomidine Pretreatment Attenuated Systemic Lipopolysaccharide-Induced Mechanical Hyperalgesia in Neonatal Rats

**FIGURE 8:**
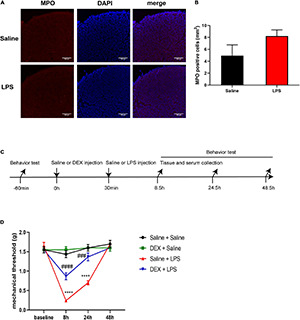
DEX pretreatment attenuated LPS-induced mechanical hyperalgesia. **(A)** Representative photomicrographs of myeloperoxidase (MPO) immunostaining in the rat spinal cord dorsal horn 8 h after saline or LPS injection. Scale bars, 100 μm. **(B)** Systemic LPS exposure did not induce obvious accumulation of MPO-positive cells 8 h after LPS injection. **(C)** Experimental design. P5-6 rat pups underwent intraperitoneal injection of saline or DEX. The baseline mechanical threshold was measured 1 h before saline or DEX injection. Thirty minutes after the injection, the rat pups were intraperitoneally injected with saline or LPS. Rat pups underwent a nociceptive behavior test 8, 24, and 48 h after LPS injection, or were sacrificed for mRNA analyses and ELISA 8 h after LPS injection. **(D)** Mechanical threshold was decreased in rats 8 and 24 h after LPS injection, and DEX significantly alleviated LPS-induced mechanical hyperalgesia. Results of panel **(B)** are expressed as the mean ± SEM, *n* = 4 animals for each group, and were analyzed by unpaired *t*-test. Results of panel **(D)** are expressed as the mean ± SEM, *n* = 12 animals for each group and were analyzed by two-way repeated-measures ANOVA followed by Bonferroni’s multiple comparisons test. ****P < 0.0001 Saline + LPS group vs. Saline + Saline group. ^###^*P* < 0.001, ^####^*P* < 0.0001 DEX + LPS group vs. Saline + LPS group.

Neutrophils are important inflammatory cells that can be upregulated in the CNS after damage and are one of the sources of pro-inflammatory cytokines (Ji et al., 2007; Steinbach et al., 2013; Alam et al., 2020). We performed the immunolabeling of neutrophils with myeloperoxidase (MPO) and investigated whether the anti-inflammatory effect of DEX might be through the inhibition of neutrophil accumulation. Although we found similar enhanced immunostaining of MPO in ischemic tissue as previously reported (Kong et al., 2014) (data not shown), to our surprise, our data indicated that there was no obvious change of MPO staining in the dorsal horn of the spinal cord between the sham control and LPS-injection 8 h (Figures 8A,B), indicating that neutrophils are not the major participator in LPS-induced spinal inflammation at this point.

We next examined the effect of DEX treatment on LPS-induced mechanical hyperalgesia by the procedure shown in Figure 8C. The baseline mechanical threshold was not significantly different among the four groups (*P* > 0.05). There was no significant difference in mechanical threshold between the Saline + Saline group and the DEX + Saline group at each time point (*P* > 0.05) (Figure 8D). The rats in Saline + LPS group showed a significant decrease in mechanical threshold at 8 and 24 h after LPS injection compared with the rats in the Saline + Saline group (8 h: 0.24 ± 0.03 vs. 1.43 ± 0.09, *P* < 0.0001; 24 h: 0.7 ± 0.05 vs. 1.6 ± 0.09, *P* < 0.0001). In contrast, DEX pretreatment significantly attenuated the LPS-induced mechanical hyperalgesia in neonatal rats as early as 8 h and had a continuous protection until the 24-h checkpoint after the LPS injection (8 h: 0.87 ± 0.09 vs. 0.24 ± 0.03, *P* < 0.0001; 24 h: 1.37 ± 0.1 vs. 0.7 ± 0.05, *P* < 0.001, Figure 8D).

## Discussion

Our study showed that a single systemic LPS injection induced acute mechanical hyperalgesia in neonatal rats. Increased levels of pro-inflammatory cytokines in serum, short-term pro-inflammatory cytokine production, and microglial activation in the spinal cord were also observed in neonatal rats that underwent LPS injection. Pretreatment with DEX significantly decreased LPS-induced inflammation, M1 microglial marker expressions, and microglial pro-inflammatory cytokine expressions, which consequentially alleviated LPS-induced mechanical hyperalgesia in neonatal rats.

As a major component of the outer membrane of gram-negative bacteria, LPS can induce pain hypersensitivity in both humans and animals (Guo and Schluesener, 2006; Karshikoff et al., 2015; Wegner et al., 2015). Intracerebral injection of LPS in neonatal rats could cause a long-lasting hyperalgesia until adulthood (Wang et al., 2011). In our study, we did find that systemic LPS injection induced an acute mechanical hyperalgesia as early as in neonates. This result was consistent with another study that a single systemic LPS injection elicited acute mechanical hyperalgesia in adult rats (Guo and Schluesener, 2006). Unlike LPS-induced hypersensitivity to mechanical stimulation, we did not find any significant changes in thermal latency after LPS injection, compared with a previous study that reported that LPS injection resulted in a reduction in both mechanical threshold and thermal latency in neonatal rats (Hsieh et al., 2018). In their research, a tail-flick test was performed to assess thermal latency, while in our study, we performed a plantar test instead. This difference may be possibly caused by the differences in nociceptive behavior assessment methods.

Systemic LPS injection can elicit inflammation in the CNS, but the accurate mechanism has not been fully elucidated. The interaction of LPS and endogenous immune cells in the CNS and the subsequent release of pro-inflammatory cytokines may contribute to LPS-induced neuroinflammation (Lund et al., 2006; Bowyer et al., 2020). Another possible mechanism is the transition of pro-inflammatory cytokines in circulation to the CNS, which activates endogenous immune cells in the CNS and triggers inflammatory responses (Banks et al., 1991; Pan and Kastin, 2002; Qin et al., 2007; Banks and Erickson, 2010; Swaroop et al., 2016; Bras et al., 2020). Consistent with previous studies (Hsieh et al., 2018, 2020), our findings clearly proved that systemic LPS injection induces the release of pro-inflammatory cytokines in the spinal cord of neonatal rats (Figures 3, 6). Previous studies have reported that LPS exposure could result in persistent inflammation in the CNS, which may influence brain function (Qin et al., 2007; O’loughlin et al., 2017; Gisslen et al., 2019). Moreover, rats injected with LPS during the neonatal period also displayed enhanced pain sensitivity later in life (Boisse et al., 2005; Zouikr et al., 2014, 2015). Thus, it is very possible that LPS can induce persistent inflammation in the spinal cord of neonatal rats which mediates central sensitization and pain hypersensitivity later in life. In our study, although our results showed that LPS exposure induced an enhanced short-term accumulation of spinal pro-inflammatory cytokines, which was resolved after 48 h, seeking a suitable experimental animal disease model should also be taken into consideration.

Spinal microglia play a key role in hyperalgesia, and the mechanism includes microglial polarization toward a pro-inflammatory (M1) phenotype and production of pro-inflammatory cytokines (Kawasaki et al., 2008; Orihuela et al., 2016; Gong et al., 2017). The pro-inflammatory cytokines, namely, IL-1β and TNF-α, can induce central sensitization either by increasing excitatory synaptic transmission or decreasing inhibitory synaptic transmission in the dorsal horn of the spinal cord (Kawasaki et al., 2008), which results in the enhancement of pain signal transmission (Ji et al., 2016). Previous studies have reported that spinal microglial activation was implicated in LPS-induced hyperalgesia (Yoon et al., 2012; Hsieh et al., 2018). In our study, we found LPS-induced upregulations of M1 microglial markers in the spinal cord. As microglial responses to a noxious stimulus also include proliferation and morphological changes, i.e., enlarged cell bodies and shortened processes (Chen et al., 2018), we measured microglial cell number and soma size in the dorsal horn of the spinal cord by using Iba1 immunostaining (Figures 4C–G). Although LPS did not induce significant changes in the number of microglial cells 24 h after LPS injection, microglia did appear to have larger cell bodies, an implication of cell activation. We also noticed a chronological order between the morphological changes in microglia and the inflammatory profiles in the spinal cord, as the expressions of M1 microglial markers and pro-inflammatory cytokines were elevated 8 h after LPS injection, while the microglial morphological changes took place 24 h after LPS injection. This finding is consistent with a prior study that microglial morphological changes were delayed compared with microglial inflammatory cytokine expression after the LPS challenge (Norden et al., 2016).

Dexmedetomidine has been widely used in pediatric clinical practice in recent years, and its anti-inflammatory effects have been increasingly studied (Ning et al., 2017; Feng et al., 2019; Li R. et al., 2020). Consistent with these studies, we found that DEX could alleviate inflammation, both in the serum and the spinal cord, in neonatal rats injected with LPS. As the inhibition of microglial activation and microglial polarization toward the M1 phenotype has contributed to the anti-inflammatory property of DEX in the CNS (Yeh et al., 2018; Gao et al., 2019; Meng et al., 2020), we next examined the effects of DEX on the expression levels of M1 microglial markers and indeed found that DEX pretreatment decreased the LPS-induced expressions of M1 microglial markers in the spinal cord of neonatal rats. To further confirm the protective effects of DEX on microglia, we sorted microglia from the spinal cord and found that DEX pretreatment decreased LPS-induced expressions of pro-inflammatory cytokines in microglia. Considering that the pro-inflammatory cytokines in circulation can transport to the CNS, which may activate microglia and trigger inflammatory responses (Banks et al., 1991; Pan and Kastin, 2002; Qin et al., 2007; Banks and Erickson, 2010; Swaroop et al., 2016; Bras et al., 2020), the anti-neuroinflammation effect of DEX in our study might be partially through the alleviation of periphery inflammation as well, which subsequently inhibits microglial activation and microglial polarization toward the M1 phenotype. Meanwhile, microglia can be beneficial (Gomes-Leal, 2012). Microglia can polarize toward the M2 phenotype and release anti-inflammatory cytokines, such as IL-10 and IL-4, under certain conditions (Orihuela et al., 2016). It has been shown that IL-10 and IL-4 could suppress pro-inflammatory responses (Marques et al., 2004). A previous study has demonstrated that blocking IL-10 activity could result in hyperalgesia (Mckelvey et al., 2015). As DEX can induce M2 microglial polarization and increase the levels of IL-10 and IL-4 in microglia (Sun et al., 2018; Gao et al., 2019; Qiu et al., 2020), it is very possible that the upregulation of microglial anti-inflammatory cytokine expressions in the spinal cord may also be implicated in the anti-inflammatory and anti-hyperalgesia effects of DEX. However, in our model, we did not detect a significant difference in the expressions of IL-10 and IL-4 in microglia 8 h after LPS injection. Moreover, there were no obvious effects of DEX on the expressions of IL-10 and IL-4 in the microglia. Thus, the effect of anti-inflammation could occur in the late stage after LPS injection.

We also noticed the inconsistency of the pro-inflammatory cytokine mRNA expressions between the sorted microglia and the spinal cord tissue, which indicated the possible involvement of other cells in spinal inflammation. Astrocytes, another type of glial cells in the CNS, have been reported to produce iNOS and CD86 as well as pro-inflammatory cytokines in response to stimulus *in vitro* (Soos et al., 1999; Cheng et al., 2020). However, astrogliosis and increase in cyclooxygenase-2 (COX-2) have been mainly found *in vivo* in the spinal cord of neonatal rats after LPS injection (Hsieh et al., 2018, 2020). A detailed study on astrocytic contribution needs to be fully investigated in the future. Neutrophils are important inflammatory cells that are upregulated in the CNS after damage and are a source of pro-inflammatory cytokines (Ji et al., 2007; Steinbach et al., 2013; Alam et al., 2020). It has been shown that neutrophils could be observed in the brain after systemic LPS injection (Zhou et al., 2009; Wu et al., 2020). Microglia can be activated and phagocytize neutrophils (Neumann et al., 2008). Therefore, we detected neutrophils in the dorsal horn of the spinal cord 8 h after LPS injection, at which time point the inflammation was drastic and DEX showed an obvious anti-inflammatory property. However, there was no obvious accumulation of neutrophils at this time point. More detailed studies on the involvement of neutrophils in LPS-induced spinal inflammation are required in the future.

In summary, we demonstrated that a single systemic LPS injection induced acute mechanical hyperalgesia, systemic inflammation, and short-term pro-inflammatory cytokine production and microglial activation in the spinal cord of neonatal rats. Pretreatment with DEX significantly decreased the spinal inflammation and alleviated the mechanical hyperalgesia induced by LPS, which is possible through the inhibition of M1 microglial polarization and microglial pro-inflammatory cytokine expression. Therefore, our finding highlights a new therapeutic target in the treatment of infection-induced hyperalgesia in neonates and preterm infants. This treatment may further prevent cognitive deficits and abnormal pain sensitivity caused by noxious stimuli and painful experiences in early life.

## Data Availability Statement

The original contributions presented in the study are included in the article/supplementary material, further inquiries can be directed to the corresponding author/s.

## Ethics Statement

The animal study was reviewed and approved by the Laboratory Animal Welfare and Ethics Committee of Shanghai Children’s Medical Center.

## Author Contributions

WW, XG, JZ, XT, and MZ designed the study. WW, HC, and YY collected the data. WW, XG, and MC analyzed the data. WW drafted the manuscript. XT and MZ reviewed, edited, and finalized the manuscript. All authors contributed to the article and approved the submitted version.

## Conflict of Interest

The authors declare that the research was conducted in the absence of any commercial or financial relationships that could be construed as a potential conflict of interest.

## Publisher’s Note

All claims expressed in this article are solely those of the authors and do not necessarily represent those of their affiliated organizations, or those of the publisher, the editors and the reviewers. Any product that may be evaluated in this article, or claim that may be made by its manufacturer, is not guaranteed or endorsed by the publisher.
